# The association of secondary hyperparathyroidism and myocardial damages in hemodialysis end-stage renal disease patients: assessed by cardiovascular magnetic resonance native T1 mapping

**DOI:** 10.1186/s12968-021-00713-8

**Published:** 2021-03-11

**Authors:** Huayan Xu, Wanlin Peng, Zhigang Yang, Yi Zhang, Chunchao Xia, Zhenlin Li, Rong Xu, Yingkun Guo

**Affiliations:** 1 Department of Radiology, West China Second University Hospital, Sichuan University, 20# South Ren min Road, Chengdu, 610041 Sichuan China; 2 Key Laboratory of Obstetric & Gynecologic and Pediatric Diseases and Birth Defects of Ministry of Education, West China Second University Hospital, Sichuan University, 20# South Ren min Road, Chengdu, 610041 Sichuan China; 3 Department of Radiology, West China Hospital, Sichuan University, 37# Guo Xue Xiang, Chengdu, 610041 Sichuan China

**Keywords:** Secondary hyperparathyroidism, End stage renal disease, Native T1 mapping, Cardiovascular magnetic resonance

## Abstract

**Background:**

Secondary hyperparathyroidism is a common complication of end-stage renal disease (ESRD), which may be associated with cardiovascular diseases. Thus, this study aimed to explore myocardial damage using non-contrast cardiovascular magnetic resonance (CMR) in ESRD patients undergoing hemodialysis and further investigate its relationship with parathyroid hormone (PTH) toxicity.

**Methods:**

Seventy-two adult ESRD patients receiving regular hemodialysis and 30 healthy subjects underwent CMR examination. Continuous CMR cine sections from the mitral valve level to the left ventricular (LV) apex in the short-axis plane, cine series of vertical two-chamber long-axis plane, and horizontal four-chamber plane were acquired. Native T1 mapping was obtained using modified Look-Locker inversion recovery (MOLLI) sequences. Native T1 values and myocardial strain were analyzed.  Immunoreactive parathyroid hormone (iPTH) was obtained from all enrolled patients.

**Results:**

Forty (55.6%) hemodialysis ESRD patients were found to have increased iPTH levels. LV ejection fraction (LVEF) of both ESRD patients with targeted and increased iPTH levels was decreased compared with healthy subjects (55.9 ± 12.0% vs. 65.0 ± 4.5%; 51.7 ± 12.8 vs. 65.0 ± 4.5%, both P < 0.05). The mean peak radial strain (PRS), peak circumferential strain (PCS), and peak longitudinal strain (PLS) were lowest in ESRD patients with increased iPTH; however, no significant difference was observed among these three groups. Segmentally, from base to apex, the native T1 of ESRD patients with increased iPTH levels tended to be higher than those with targeted iPTH and healthy subjects (all P < 0.05). In ESRD patients with targeted iPTH, both native T1 of basal and middle segments were significantly higher than normal subjects (basal, 1304 ± 41 ms vs. 1238 ± 36 ms, P = 0.001; middle, 1300 ± 43 ms vs. 1242 ± 50 ms, P < 0.001). Comparing global native T1 values in the three groups, ESRD patients with targeted and increased iPTH level showed increased native T1 (1305 ± 41 ms vs. 1251 ± 49 ms, P = 0.001; 1334 ± 40 ms vs. 1251 ± 49 ms, P < 0.001, respectively). Native T1 values of the basal segment and global native T1 were moderately associated with iPTH (r = 0.4, P < 0.001; r = 0.5, P < 0.001). Multiple linear regression analysis showed that global native T1 values (beta = 1.0, P = 0.01) were independently associated with iPTH.

**Conclusions:**

Elevated iPTH level was associated with and was an independent risk factor for myocardial damage in ESRD patients undergoing maintenance hemodialysis.

*Trial registration: *Chinese Clinical Trial Registry (http://www.chictr.org.cn/index.aspx) ChiCTR-DND-17012976, 13/12/2017, retrospectively registered.

## Background

Secondary hyperparathyroidism is a common complication of end-stage renal disease (ESRD) with maintenance hemodialysis, which begins in the earlier stages of chronic renal insufficiency and is shown to be deteriorated with declining renal function. Hyperphosphatemia, hypocalcemia, decreased calcium, and vitamin D receptor expression, 1,25-dihydroxyvitamin D3 deficiency, and parathyroid hormone (PTH) resistance may partly play a role in the secondary hyperparathyroidism pathogenesis [1]. Previous studies have indicated that hyperparathyroid disorders, including primary and secondary hyperparathyroidism, are risk factors for cardiovascular morbidity and mortality [2, 3]. Hagström et al. demonstrated that high PTH levels can predict nonischemic heart failure (HF), and higher plasma parathyroid levels were significantly correlated with the advanced New York Heart Association (NYHA) level [5]. Meanwhile, plasma PTH level may have a positive value in the diagnostic criteria of HF [6, 7]. Several mechanisms may contribute to HF in high PTH circumstances, such as specific vascular endothelial dysfunction promotion and atherosclerosis-induced cardiac ischemia, or direct detrimental myocardial effects such as myocyte hypertrophy and fibrosis [5]. Some prior studies have deduced the association between elevated PTH levels and myocardial damage or even HF in patients with chronic renal insufficiency by investigating the B-type natriuretic peptide level, NYHA, myocardial infarction, or HF history, or in animal models. However, no direct evidence was acquired to prove a relationship between prolonged exposure to elevated PTH and myocardial damage measured by noninvasive imaging methods such as cardiovascular magnetic resonance (CMR). Myocardial edema, diffused myocardial fibrosis, and left ventricular (LV) deformation may be key pathogenesis of ESRD-related myocardiopathy [8–12]. Thus, novel imaging biomarkers that can reliably and precisely measure pathological cardiac changes that are strongly linked to cardiac outcomes are needed. CMR late gadolinium enhancement (LGE) is a common imaging marker for evaluating myocardial fibrosis. However, gadolinium-based CMR contrast agents may cause complication of nephrogenic systemic fibrosis in patients with ESRD [13]. Hence, gadolinium is relative contraindicated for ESRD patients [14]. CMR native T1 mapping is a novel and non-contrast technique that can quantitatively measure myocardial fibrosis or myocardial edema [14], and myocardial strain by tissue tracking can reflect LV deformation, which may provide direct evaluation tools to assess the myocardial damage in ESRD patients. Therefore, this research was to explore cardiac involvement by examining CMR native T1 mapping and myocardial strain and further investigate the relationship between uremic myocardiopathy and PTH toxicity in ESRD hemodialysis patients.

## Methods

### Study subjects

A total of 100 adult ESRD hemodialysis patients were prospectively recruited from September 2017 to July 2019. The inclusion criteria of ESRD patients were deteriorating renal function or kidney damage for more than 3 months and stage 5 chronic kidney disease (CKD) with an estimated glomerular filtration rate (eGFR) of < 15 mL/min/1.73 m^2^ [15]. The exclusion criteria included a clinical history of diabetes and primary hypertension-induced ESRD (n = 8), presence of echocardiography and clinical history demonstrating congenital cardiac disease or primary cardiomyopathy (n = 0), X-ray angiography verified coronary artery disease (n = 5); incomplete CMR T1 mapping data (n = 3), and poor CMR images (including poor cine images, n = 4; poor T1mapping images, n = 8) and those patients with contradictions of CMR (n = 0). After exclusion, 72 ESRD patients were enrolled. All patients with ESRD underwent hemodialysis regularly (twice weekly). All CMR scans were performed the day before dialysis. In addition, we enrolled 30 healthy individuals who had no chronic systemic diseases, diabetes mellitus, hypertension, any cardiovascular diseases, family history of cardiovascular disease, or all causes of renal diseases. All patients and healthy controls underwent CMR imaging.

Fasting venous blood from patients with ESRD was drawn for routine blood examination, biochemical indicators, and uremic toxins. The eGFR of each ESRD patient was calculated with serum creatinine using the CKD-Epidemiology Collaboration equation [16]. All patients were treated symptomatically. Correction of hypocalcemia and hyperphosphatemia was performed if hypocalcemia and hyperphosphatemia occurred. Active vitamin D was used to reduce PTH appropriately. Immunoreactive PTH (iPTH) was also obtained using the Allegro method. ESRD patients were classified into two groups by reaching the therapy target of iPTH or not. The therapeutic plasma iPTH target is 150–300 pg/mL according to the Kidney Disease Improving Global Outcomes (KDIGO) guidelines [17]. Thus, ESRD patients were divided into patients with targeted (iPTH < 300 pg/mL) and increased iPTH (iPTH > 300 pg/mL) cohorts.

### CMR protocol

All patients were imaged supine on a 3 T CMR scanner (Skyra, Siemens Healthineers, Erlangen, Germany) with an 18-element body phased array coil. Electrocardiogram (ECG) and breath-hold triggers were required for each patient to acquire high-quality images. Stacks of retrospectively gated cine balanced steady state free precession (bSSFP) sequences (temporal resolution 43 ms, TR 39.1 ms, TE 1.4 ms, slice thickness 8.0 mm, field-of-view 280.4 × 340.0 174 × 208, flip angle 60°) from the base to apex were obtained in the short-axis (SAx) plane as well as the cine series of vertical two-chamber long-axis plane and horizontal four-chamber plane. Native T1 mapping was performed by using a modified Look-Locker inversion recovery (MOLLI) sequences, the scanning model of native T1 MOLLI sequence was 5(3)3 (TE 1.1 ms, TR 346.6 ms, field of view 306.6 × 360.0 218 × 256, slice thickness 5.00 mm, flip angle 35°), and B0 shimming (targeted cardiac mode) was used to minimize the off-resonance artifacts. The basal, middle, and apical SAx slices of native T1 mapping were acquired. Eight echoes of native T1 source imaging and color-coded native T1 maps were generated for analysis.

### Image analysis

**Fig. 1:**
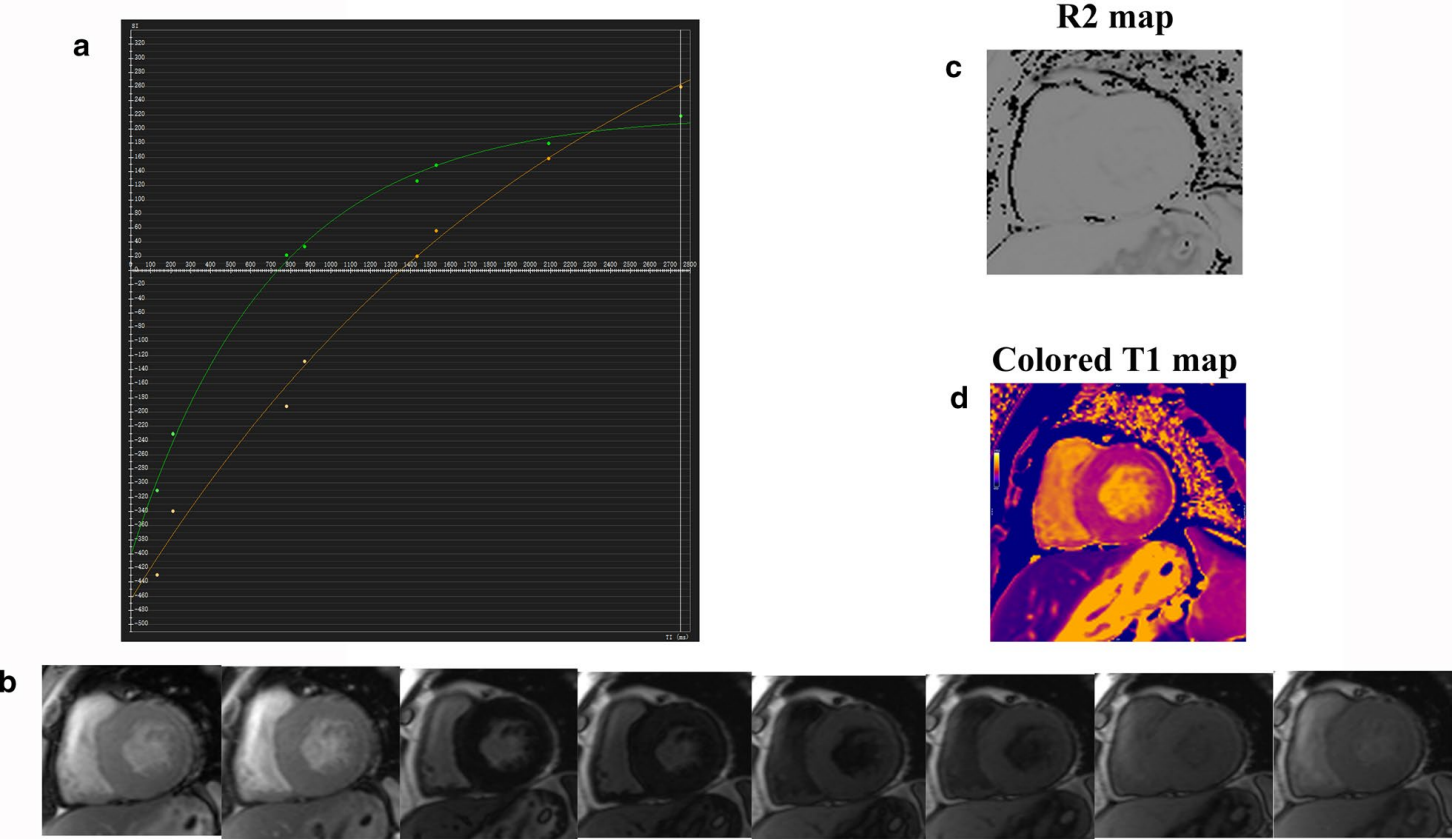
Inversion recovery curves of T1 mapping. The inversion recovery curve (**a**) and R2 map are generated from the T1-weighted source images (**b**). The signal intensity in each pixel of T1-weighted source images is used to fit the colored T1 (**d**) and R2 maps (**c**) that are generated

**Fig. 2:**
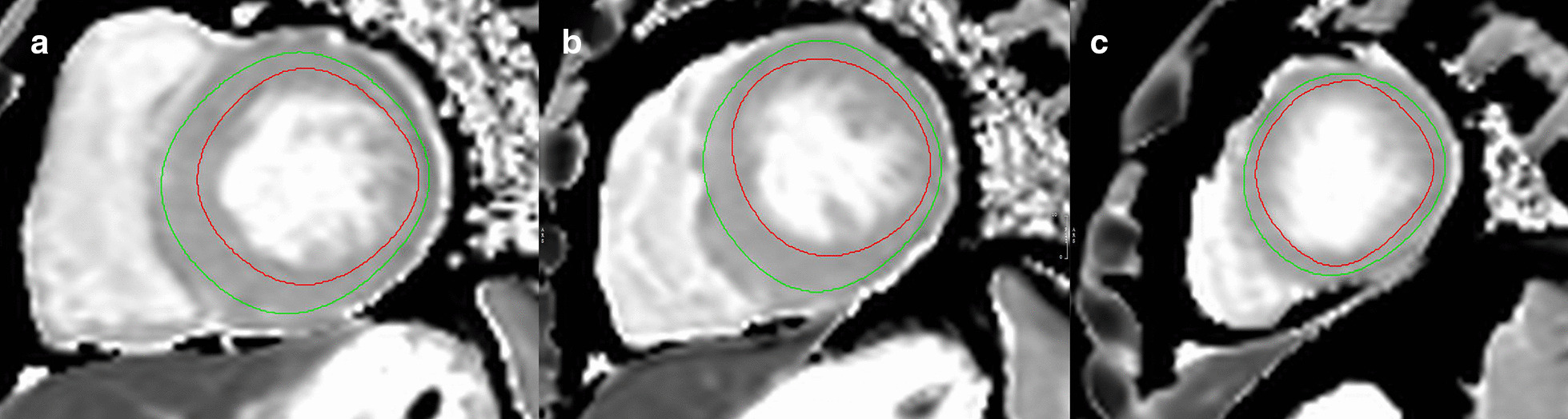
Endocardial (red curve) and epicardial (green curve) delineations for the acquisition of native T1 value in basal (**a**), middle (**b**), and apical (**c**) segments

Post-processing of all images was conducted using offline commercial software (cvi42, version 5.9.3, Circle Cardiovascular Imaging, Calgary, Alberta, Canada). For cardiac function assessment, LV function parameters (LV ejection fraction [LVEF], end-diastolic volume [LVEDV], end-systolic volume [LVESV], and stroke volume [SV], LV mass) were measured and automatically computed by manually tracing the endo- and epicardial borders on the stacks of SAx cine images at the end-diastolic and systolic phases. Blood volume and papillary muscles were excluded. For myocardial strain measurement, sets of SAx slices and horizontal four-chamber and long-axis two-chamber cine images were loaded into the tissue tracking module. Afterwards, endo- and epicardial boundaries were manually drawn on the LV end-diastolic phase of all series, and the LV extending from the mitral valve to the apex was defined in both the four-chamber and two-chamber series. Subsequently, the anterior and posterior SAx reference points were placed on the insertion point of the septum and LV free wall on the SAx plane. Blood volume and papillary muscles were also excluded. Myocardial strain parameters including peak radial strain (PRS), peak circumferential strain (PCS), and peak longitudinal strain (PLS) directions were calculated. For segmental native T1 value measurements, native T1 source images were loaded into the T1 calculation module, endo- and epicardial boundaries were manually traced, and T1 recovery curve, native T1 color maps, R2 maps, and native R2 values were generated (Fig. 1). Native T1 color maps were directly uploaded into the map analysis parts of this module, and native T1 values of basal, middle, and apical segments were automatically computed (Fig. 2). Then we manually draw the endo- and epicardial borders on the grayscale images as close to the myocardium layer as possible to avoid the effect of blood pool or epicardial and pericardial fat. Global native T1 values across the slices were also automatically generated after the native T1 values of basal, middle, and apical SAx segments were all acquired [19].

### Reproducibility of myocardial strain and native T1 value

To assess intraobserver and interobserver variabilities, data from 25 cases were randomly selected from both healthy and ESRD subjects. Intraobserver variability was assessed by comparing the myocardial strain parameters and native T1 measured by the same observer after an interval of 2 weeks between measurements. The interobserver variability was obtained by two independently experienced and double-blinded observers.

### Statistical analysis

All statistical analyses were performed using SPSS (version 21.0, Statistical Package for the Social Sciences, International Business Machines, Inc., Armonk, New York, USA) or GraphPad Prism (version 7.00, Graph-Pad Software, La Jolla, California, USA). The Shapiro–Wilk test was used to examine the normality of data. Data was presented as the mean ± SD or median (quartile), which refers to the normality result. The Mann–Whitney U test and independent t-test were used to compare the characteristics between the healthy and ESRD groups according to the data’s characteristics. Comparisons among multiple groups were performed by one-way analysis of variance with post hoc Bonferroni correction. Bivariate correlations were calculated using the Pearson or Spearman method as appropriate. Multiple linear regression was performed to detect the CMR correlates of iPTH in myocardial damage. The reproducibility of myocardial strain and native T1 was assessed using the intraclass correlation coefficient (ICC). Two-tailed P < 0.05 were considered statistically significant.

## Results

### Baseline characteristics of ESRD patients

The demographic and biochemical indicators of the 72 ESRD patients and 30 healthy controls are shown in Table 1 with no significant differences found in age, sex, height, and weight (all P > 0.05). The duration of renal insufficiency in the ESRD patients was 12–228 months, and the hemodialysis time was 9–228 months. A total of 10 (13.9%) polycystic kidney disease, 52 (72.2%) primary glomerular nephropathy, 4 (5.6%) vasculitis, and 6 (8.3%) genitourinary tuberculosis accounted for the major ESRD causes.

**Table 1:** Basal characteristics of healthy and end-stage renal disease (ESRD) hemodialysis patients with targeted and with increased immunoreactive parathyroid hormone (iPTH)

	Healthy subjects (n = 30)	ESRD with targeted iPTH (n = 32)	ESRD with increased iPTH(n = 40)	P
Age (years)	48.1 ± 11.6	58.2 ± 13.9	57.4 ± 15.0	0.06
Gender (Male, n)	12(40.0%)	12(37.5%)	15(37.5%)	0.54
Height (cm)	152.8 ± 7.9	154.8 ± 23.1	156.5 ± 6.5	0.41
Weight (kg)	53.6 ± 6.6	50.3 ± 3.2	53.3 ± 9.6	0.47
Heart rate (beats/min)	71 ± 9	81 ± 12	78 ± 13	0.08
Duration of dialysis (M)	N/A	12–132	9–228	0.06
Duration of renal insufficiency (M)	N/A	12–156	12–228	0.06
Systolic BP	N/A	136 ± 26	142 ± 20	0.34
Diastolic BP	N/A	82 ± 13	89 ± 17	0.24
Causes of ESRD				
Polycystic kidney disease (n, %)	N/A	4(12.5%)	6(15.0%)	N/A
Primary glomerular nephropathy (n, %)	N/A	23(44.3%)	29(55.8%)	N/A
Vasculitis (n, %)	N/A	1(3.1%)	3(7.5%)	N/A
Genitourinary tuberculosis (n, %)	N/A	2(6.3%)	4(10.0%)	N/A

Values are mean ± SD or n (%)

*M* months, *BP* blood pressure, *ESRD* End-stage renal disease, *CKD* chronic kidney disease, *iPTH* immunoreactive parathyroid hormone

Biochemical results are listed in Table 2. Uremic toxins, such as creatinine and urea, in both ESRD patients with targeted and increased iPTH levels were both increased when compared with the normal ranges; however, no difference was found between the two groups. The eGFR of ESRD patients representing the renal glomerular filtration capacity is extremely lower than that of the normal reference. Thirty-two (44.4%) ESRD patients had reduced calcium  and increased magnesium levels compared to the normal reference. Furthermore, 35 (48.6%) patients had serum phosphorus abnormality. The iPTH range is 44–2964 (mean, 547 ± 577). Moreover, 32 (44.4%) patients were found to have targeted iPTH (normal range 150–300 pg/mL), and 40 (55.6%) ESRD patients still had increased iPTH levels. However, no difference in the biochemical results, such as hemoglobin, hematocrit, uremic toxins, eGFR, Ca^2+^, and Mg^2+^, were found between ESRD patients with targeted and increased iPTH. Otherwise, all ESRD patients were orally administered calcium acetate management.

**Table 2:** The biochemical results

	ESRD with targeted iPTH (n = 32)	ESRD with increased iPTH (n = 40)	P
Hemoglobin (g/L) (n range 110–160)	96 ± 13	98 ± 10	0.64
Hematocrit (%) (normal range 37–54)	0.3 ± 0.1	0.3 ± 0.1	0.17
eGFR (mL/min per 1·732 m^2^) (normal range 80–120)	6 ± 2	5 ± 3	0.25
Creatinine (umol/l) (normal range 53.0–140.0)	1020 ± 1658	849 ± 303	0.51
Uric acid (umol/L) (normal range 240–490)	412 ± 120	397 ± 113	0.47
Urea (mmol/L) (normal range 3–8)	21 ± 7	23 ± 9	0.38
iPTH (pg/mL) (normal range 150–300)	188 ± 71	832 ± 642	0.00
Calcium (mmol/L) (normal range 2.1–2.7)	2.1 ± 0.2	2.2 ± 0.3	0.80
Magnesium (mmol/L) (normal range 0. 7–1.0)	1.0 ± 0.1	1.0 ± 0.1	0.89
Serum phosphorus (mmol/L) (normal range 1.5–2.1)	1.5 ± 0.5	1.8 ± 0.6	0.06

Values are mean ± SD

*iPTH* immunoreactive parathyroid hormone, *eGFR* estimated glomerular filtration rate

### Comparison of myocardial damages assessed using CMR

**Fig. 3:**
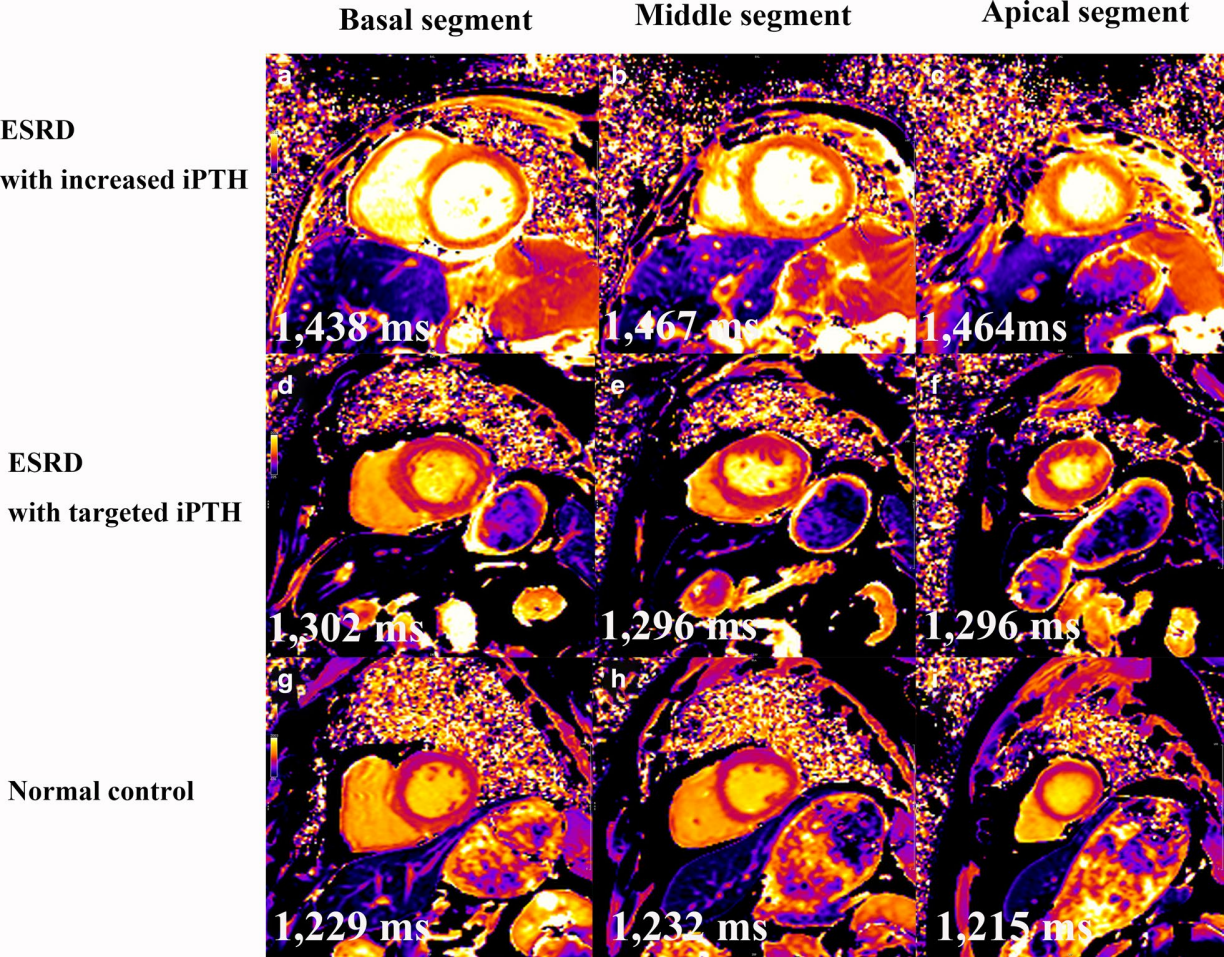
Represented cases of a native T1 map in end-stage renal disease (ESRD) patients. The first row shows that the native T1 values of basal (**a** native T1 = 1438 ms), middle (**b** native T1 = 1467 ms), and apical (**c** native T1 = 1464 ms) segments of a patient with increased iPTH (iPTH = 931 pg/mL) are higher than those with targeted iPTH [(second row, iPTH = 91 pg/mL); basal (**d**), native T1 = 1302 ms; middle (**e**), native T1 = 1296 ms; apical (**f**), native T1 = 1296 ms] and normal control (third row) [basal (**g**), native T1 = 1229 ms; middle (**h**), native T1 = 1232 ms; apical (**i**), native T1 = 1215 ms]

For LV function (Table 3), the LVEF of both ESRD patients with targeted and increased iPTH was found to be decreased comparing with that in healthy subjects (56 ± 12% vs. 65 ± 5%; 52 ± 13 vs. 65 ± 5%, both P < 0.05). Although no significant difference was found between ESRD patients with targeted and increased iPTH cohorts, the LVEF in patients with increased iPTH tended to be lower than those with targeted iPTH. For other LV function parameters, only the LVESV of ESRD patients with increased iPTH was enlarged. LVEDV and SV did not differ significantly between the healthy and the two ESRD groups. LV parameters indexed to the body surface area showed the same tendency. For myocardial strain (Table 3), the mean PRS, PCS, and PLS were lowest in ESRD patients with increased iPTH levels. Nevertheless, no significant differences were observed among the three groups. For native T1 mapping assessing myocardial damage (Fig. 3), segmental and global native T1 values were assessed and compared. Segmentally, from the basal to apical segment, the native T1 values of ESRD patients with increased iPTH tended to be higher than those with targeted iPTH and healthy individuals (all P < 0.05). In ESRD patients with targeted iPTH, the native T1 of the basal and middle segments were significantly higher than that in the normal subjects (1304 ± 41 ms vs. 1238 ± 36 ms; 1300 ± 43 ms vs. 1242 ± 50 ms, both P < 0.001), and the apical segment native T1 values had no difference in both normal and ESRD patients with targeted iPTH individuals. Comparing the global native T1 values in the three groups, ESRD patients with targeted and increased iPTH showed increased native T1 values (1305 ± 41 ms vs. 1251 ± 49 ms, P = 0.001; 1334 ± 40 ms vs. 1251 ± 49 ms, both P < 0.001). Meanwhile, ESRD patients with increased iPTH levels were also higher than the targeted iPTH ones for the global native T1 values (1334 ± 40 ms vs. 1305 ± 41 ms, P = 0.01).

**Table 3:** LV function, myocardial strain and native T1 value of ESRD with targeted iPTH and with increased iPTH

	Normal subjects (n = 30)	ESRD with targeted iPTH (n = 32)	ESRD with increased iPTH (n = 40)
LV function			
EF (%)	65.0 ± 4.5	55.9 ± 12.0*	51.7 ± 12.8*
EDV (mL)	117 ± 31	128 ± 44	134 ± 50
ESV (mL)	45 ± 13	60 ± 44	69 ± 47*
SV (mL)	79 ± 17	67 ± 18	65 ± 18
LV mass (g)	52 ± 17	85 ± 31*	90 ± 32*
EDV/BSA (mL/mm^2^)	72.3 ± 20.1	83.3 ± 26.4	84.8 ± 36.3
ESV/BSA (mL/m^2^)	260.0 ± 5.5	38.9 ± 26.4	43.8 ± 34.4 *
SV/BSA (mL/m^2^)	48.6 ± 11.2	44.2 ± 11.6	40.3 ± 10.9*
Mass/BSA (g/m^2^)	32.2 ± 11.4	54.4 ± 19.0*	56.3 ± 19.0*
Myocardial strain parameters			
PRS	43.0 ± 9.8	43.2 ± 14.9	37.6 ± 15.7
PCS	− 18.3 ± 2.1	− 17.8 ± 4.2	− 16.8 ± 5.0
PLS	− 16.5 ± 2.4	− 15.7 ± 3.7	− 14.8 ± 4.4
Native T1 value			
Basal segment	1238 ± 36	1304 ± 41*	1330 ± 43*#
Middle segment	1242 ± 50	1300 ± 43*	1323 ± 43*#
Apical segment	1280 ± 65	1339 ± 86	1360 ± 74*
Global	1251 ± 49	1305 ± 41*	1334 ± 40*#

*LV* left ventricular, *EDV* end-diastolic volume, *ESV* end-systolic volume, *EF* ejection fraction, *SV* stroke volume, *PRS* peak radial strain, *PCS* peak circumferential strain, *PLS* peak longitudinal strain directions, *BSA* body surface area. Other abbreviations are the same as Tables 1 and 2

*P < 0.05 vs. normal group; ^#^P < 0.05 vs. comparison with ESRD with targeted iPTH

### The independent myocardial damage factors associated with iPTH

The correlation coefficients of iPTH for secondary hyperparathyroidism evaluation are presented in Table 4. LVEF was negatively correlated with iPTH (r = − 0.3, P = 0.01), while LVEDV and LVESV were positively correlated with iPTH (r = 0.2, P = 0.04; r = 0.3, P = 0.003, respectively). From the myocardial deformation aspect, both PRS and PCS were significantly correlated with iPTH (r = − 0.2, P = 0.04; r = 0.2, P = 0.04). By analyzing the segmental and global native T1, we found that the native T1 of the basal segment (r = 0.4, P < 0.001) and the global native T1 (r = 0.5, P < 0.001) were moderately associated with iPTH, while the native T1 of the middle segment (r = 0.4, P = 0.001) showed mild correlation. Multiple linear regression analysis showed that the native T1 of global hearts (beta = 1.0, P = 0.01) were independently associated with iPTH, whereas myocardial strain parameters demonstrated no independent relationship with iPTH after multiple linear regression analysis.

**Table 4:** CMR correlates of iPTH by bivariate correlation analysis and multiple linear regression

	Bivariate correlation	Multiple linear regression	
	R	Beta	P
LV function parameters			
EF (%)	− 0.3*	− 0.1	0.73
EDV (mL)	0.2*	0.2	0.72
ESV (mL)	0.3*	0.02	0.97
SV (mL)	0.02	N/A	N/A
Myocardial strain parameters			
PRS (%)	− 0.2*	0.3	0.28
PCS (%)	0.2*	0.1	0.42
PLS (%)	0.2	N/A	N/A
Native T1 values			
Basal segment (ms)	0.4*	− 0.02	0.92
Middle segment (ms)	0.4*	− 0.5	0.09
Apical segment (ms)	0.2	N/A	N/A
Global (ms)	0.5*	1.0	0.01

All abbreviations were the same as Tables 1–3

*P < 0.05

### Reproducibility and feasibility of myocardial strain and native T1 value

LV basal, middle, and apical myocardial native T1 showed excellent interobserver ICC (ICC = 0.994, 0.997, and 0.998, respectively) and intraobserver agreement (ICC = 0.993, 0.995, and 0.997, respectively). For the global myocardial native T1, the interobserver ICC was 0.999 (95% CI 0.996–1.000), and the intraobserver ICC was 0.998 (95% CI 0.993–0.999). Myocardial strain parameters also showed excellent interobserver ICC (PRS, 0.997; PCS, 0.997; PLS, 0.970) and intraobserver ICC (PRS, 0.917; PCS, 0.991; PLS, 0.958).

## Discussion

As a worldwide public health problem due to its association with multiple comorbidities, CKD is highly associated with cardiovascular events, mortality, and high medical cost burden [19]. CKD morbidity ranged from 8 to 16% in the population [20]. Projections from the Global Health Observatory suggest that the CKD mortality rate will continue to increase from 12.2 to 14 deaths per 100,000 people by the year 2030 [21]. Although several studies on various populations have reported that low eGFR and high uremic toxicity factors are associated with cardiovascular diseases, cardiovascular mortality was still about twice higher in patients with stage 3 CKD and three times higher with stage 4 CKD. Additionally, the risk of HF is approximately doubled in patients with eGFR < 60 mL/min/1.73 m^2^ [22–24]. Cardiovascular mortality is estimated to be significantly higher in people with an eGFR < 60 mL/min/1.73 m^2^ [22]. Cardiovascular risks cause most of the deaths in ESRD patients with severe renal deficiency. Multiple risk factors account for cardiovascular disease in CKD patients. In patients with severe CKD, besides the traditional risk factors such as hypertension, dyslipidemia, Na^+^ overload, Ca^2+^ and serum phosphorus abnormalities, and chronic inflammation, cardiovascular disease in CKD may also be driven by specific risk factors including anemia and malnutrition, hormonal imbalances, soft tissue calcification, erythropoietin resistance, renal replacement therapy (RRT)-related electrolyte imbalance, and high PTH level. In all the risk factors, secondary hyperparathyroidism has been linked with mineral bone disorders as well as increased cardiovascular mortality, which may be critical factors for uremic cardiomyopathy. PTH influences LV function in chronic hemodialysis patients, and plasma PTH reduction is beneficial to the uremic heart [25]. PTH lowering has been focused on as a treatment target for decades, and the effect of high PTH level on ESRD has been in research for a long time [26]. Most of the previous studies focused on the association of PTH and mineral bone disease; however, few studies have mentioned that secondary hyperparathyroidism may contribute to the cardiovascular complications of CKD. Van et al. found that higher PTH concentrations may be associated with the increased risk of cardiovascular events through a meta-analysis [27]. PTH may contribute to four major cardiovascular effects, including contractile disturbance, cardiomyocyte hypertrophy, and cardiac interstitial fibrotic and vasodilator effects. Although only the animal model by parathyroidectomy and invasive pathological examination demonstrated these correlations, very little detail and direct evidence about the inner connection of increased PTH and cardiac changes by noninvasive methods in CKD patients have been previously demonstrated.

In our study, iPTH levels in ESRD patients were much higher than the normal range. Ca^2+^ and serum phosphorus abnormalities were also found in the ESRD cohort. The iPTH of 32 patients was within the normal range by routine calcitriol and hemodynamic dialysis therapy. We found that LVEF was decreased, but still preserved in ESRD patients with either targeted iPTH or increased iPTH levels. Although no myocardial strain deterioration was detected in both ESRD cohorts, on normal iPTH, native T1 of the basal and middle segments in the LV were significantly increased. The global native T1 in subjects with both targeted and increased iPTH was higher than that in normal subjects, which may lead to the assumption that myocardial tissue deterioration happens prior to myocardial deformation. Previous studies have reported LV mechanical dyssynchrony and microvascular dysfunction detected by single-photon emission computed tomography (SPECT), and regadenoson SPECT myocardial perfusion imaging also provided a significant prognostic value in ESRD patients [28–31]. However, SPECT, especially regadenoson SPECT, has some limitations due to the utilization of vasodilators and contrast in ESRD patients with reduced clearance and longer exposure to the drug and contrast [32]. For the CMR technique, some studies have shown that native T1 assessed by CMR acquires samples from the longitudinal magnetization perturbation and extracts the real myocardial T1 without gadolinium contrast [33, 34]. Thus, native T1 mapping is currently explored as a relatively precise diagnostic modality in a wide range of heart diseases, including diffuse myocardial tissue lesions, such as ESRD patients [33]. Conversely, gadolinium increases the risk of nephrogenic systemic fibrosis making non-contrast native T1 mapping more suitable for CKD patients. Increased native T1 account for a few characterizing tissues, such as edema, inflammation, and fibrosis, affecting the interstitial space [35]. Many studies have demonstrated that native T1 has shown an important prognostic significance in discriminating disease, especially in those with diffuse myocardial damage [36]. Thus, compared with SPECT, native T1 may provide more information, for instance edema, inflammation, or fibrosis, besides the myocardial blood flow, and non-utilization of contrast is safer for ESRD patients.

CKD contributing to decreased cardiac function, cardiac hypertrophy, and increased risk of adverse cardiovascular events is referred to as chronic renocardiac syndrome. Diffuse interstitial edema, inflammation, and fibrosis always occur in CKD patients, which can manifest as increased native myocardial T1 times [37]. We found that native T1 values were higher in patients with targeted and increased iPTH, and increased iPTH values seem more serious. Interestingly, native T1 mapping detecting myocardial damage was significantly associated with and demonstrated to be significant correlates of iPTH in this study, indicating that myocardial damage does exist in ESRD patients. Moreover, secondary hyperparathyroidism may indeed contribute to uremic myocardial changes. This result was consistent with the previous results of animal experiments on the increased PTH-induced cardiac fibrosis [38, 39]. Additionally, 40 patients presented with persistently high iPTH levels, revealing that treatment with conventional hemodialysis and medications seems limited. Meanwhile, the severity of secondary hyperparathyroidism increases with the decline in renal function. iPTH levels > 50 pg/mL in patients with CKD stages 3 and 4 are associated with an escalating combined risk of death or RRT [40]. In ESRD patients with targeted iPTH, native T1 is also increased. Thus, early and dynamic detection of myocardial damage by noninvasive imaging tools and early treatment of secondary hyperparathyroidism are particularly important.

### Limitations

Although this research has demonstrated that increased iPTH levels were associated with myocardial damage in ESRD patients evaluated by CMR native T1, some limitations still exist. First, all enrolled patients in this study had ESRD and lacked the effect of iPTH on the myocardium in early-stage CKD. Second, we could not acquire an endocardial biopsy. Thus, direct association of native T1 with histologic abnormalities is lacking. Since several studies have demonstrated that native T1 plays a critical role in measuring myocardial edema and fibrosis [34], T2 mapping for the detection of edema may be further analyzed to elucidate the pathology behind increased T1 values, whether it is due to edema or fibrosis. In addition, no significant difference in myocardial strain was found in both ESRD groups and the normal group; however, the PRS of ESRD patients with targeted iPTH was found to be slightly increased, while the other parameters showed a decreasing tendency. Since multiple factors may cause the different appearance of myocardial strain in ESRD patients [41], studies with large sample sizes and different stages of CKD are needed to further explore the evolution progress of myocardial strain.

## Conclusions

Myocardial damage was found to be increased in ESRD patients with both normal and increased iPTH levels following conventional therapy. In addition, consistently high iPTH levels may be independently associated with myocardial damage in ESRD patients.

## Data Availability

The datasets used and/or analyzed during the current study are available from the corresponding author on reasonable request.

## Abbreviations

bSSFP: Balanced steady state free precession
CKD: Chronic kidney disease
CMR: Cardiovascular magnetic resonance
ECG: Electrocardiogram
EDV: End-diastolic volume
eGFR: Estimated glomerular filtration rate
ESRD: End-stage renal disease
ESV: End-systolic volume
HF: Heart failure
iPTH: Immunoreactive parathyroid hormone
KDIGO: Kidney Disease Improving Global Outcomes
LGE: Late gadolinium enhancement
LV: Left ventricle/left ventricular
LVEDV: Left ventricular end-diastolic volume
LVEF: Left ventricular ejection fraction
LVESV: Left ventricular end-systolic volume
MOLLI: Modified Look-Locker inversion recovery
NYHA: New York Heart Association
PCS: Peak circumferential strain
PLS: Peak longitudinal strain
PRS: Peak radial strain
PTH: Parathyroid hormone
RRT: Renal replacement therapy
SAx: Short axis
SPECT: Single photon emission computed tomography
SV: Stroke volume

## References

[1] Boer DIH (2002). The Severity of Secondary Hyperparathyroidism in chronic renal insufficiency is GFR-dependent, race-dependent, and associated with cardiovascular disease. J Am Soc Nephrol.

[2] Slinin Y, Foley RN, Collins AJ (2005). Calcium, phosphorus, parathyroid hormone and cardiovascular disease in hemodialysis patients: the usrds waves 1, 3 and 4 study. J Am Soc Nephrol.

[3] Ganesh SK, Stack AG, Levin NW, Hulbert-Shearon T, Port FK (2001). Association of elevated serum po(4), ca x po(4) product and parathyroid hormone with cardiac mortality risk in chronic hemodialysis patients. J Am Soc Nephrol.

[4] Van Ballegooijen AJ, Reinders I, Visser M, Brouwer IA (2013). Parathyroid hormone and cardiovascular disease events: a systematic review and meta-analysis of prospective studies. Am Heart J.

[5] Hagström E, Ingelsson E, Sundström J, Hellman P, Larsson TE, Berglund L, Melhus H, Held C, Michaëlsson K, Lind L, Arnlöv J (2010). Plasma parathyroid hormone and risk of congestive heart failure in the community. Eur J Heart Fail.

[6] Gangyong WU (2010). Predict value of plasma parathyroid hormone in patients of congestive heart failure. J Clin Cardiol.

[7] Sugimoto T, Dohi K, Onishi K, Watanabe K, Sato Y, Sugiura E, Nakamori S, Nakajima H, Nakamura M, Ito M (2013). Interrelationship between haemodynamic state and serum intact parathyroid hormone levels in patients with chronic heart failure. Heart.

[8] Chang JM, Chen SC, Huang JC, Su HM, Chen HC (2014). Anemia and left ventricular hypertrophy with renal function decline and cardiovascular events in chronic kidney disease. Am J Med Sci.

[9] López B, González A, Hermida N, Laviades C, Díez J (2008). Myocardial fibrosis in chronic kidney disease: potential benefits of torasemide. Kidney Int Suppl.

[10] Naito K, Anzai T, Yoshikawa T (2008). Impact of chronic kidney disease on postinfarction inflammation, oxidative stress, and left ventricular remodeling. J Card Fail.

[11] Cottone S, Lorito MC, Riccobene R (2008). Oxidative stress, inflammation and cardiovascular disease in chronic renal failure. J Nephrol.

[12] Fatema K, Hirono O, Takeishi Y (2002). Hemodialysis improves myocardial interstitial edema and left ventricular diastolic function in patients with end-stage renal disease: noninvasive assessment by ultrasonic tissue characterization. Heart Vessels.

[13] Bogin E, Massry SG, Harary I (1981). Effect of parathyroid hormone on rat heart cells. J Clin Invest.

[14] Kribben A, Witzke O, Hillen U, Barkhausen J, Daul AE, Erbel R (2009). Nephrogenic systemic fibrosis: pathogenesis, diagnosis, and therapy. J Am Coll Cardiol.

[15] Levey AS, Coresh J (2012). Chronic kidney disease. Lancet.

[16] Kunihiro M, Bakhtawar KM, Mark W, Jonathan RE, Tazeen HJ, Sun HJ, Kevan RP, Anoop S, David HS, Marcello T, David GW, Wen CP, Ron TG, Brenda RH, Andrew SL (2012). Comparison of risk prediction using the CKD-EPI equation and the MDRD study equation for estimated glomerular filtration rate. JAMA.

[17] Kidney Disease: Improving Global Outcomes (KDIGO) CKD-MBD Work Group. KDIGO clinical practice guideline for the diagnosis, evaluation, prevention, and treatment of Chronic Kidney Disease-Mineral and Bone Disorder (CKD-MBD) Kidney Int 113:S1–130; 2009 10.1038/ki.2009.188 19644521

[18] Schulz-Menger J, Bluemke DA, Bremerich J, Flamm SD, Fogel MA, Friedrich MG, Kim RJ, von Knobelsdorff-Brenkenhoff F, Kramer CM, Pennell DJ, Plein S, Nagel E (2020). Standardized image interpretation and post-processing in cardiovascular magnetic resonance - 2020 update : Society for Cardiovascular Magnetic Resonance (SCMR): Board of Trustees Task Force on Standardized Post-Processing. J Cardiovasc Magn Resonan.

[19] Wang WH, Chen LW, Lee CC, Sun CY, Shyu YC, Hsu HR, Chien RN, Wu IW (2017). Association between parathyroid hormone, 25 (OH) vitamin D, and chronic kidney disease: a population-based study. Biomed Res Int.

[20] Gansevoort RT, Correa-Rotter R, Hemmelgarn BR, Jafar TH, Heerspink HJ, Mann JF, Matsushita K, Wen CP (2013). Chronic kidney disease and cardiovascular risk: epidemiology, mechanisms, and prevention. Lancet.

[21] Webster AC, Nagler EV, Morton RL, Masson P (2017). Chronic kidney disease. Lancet.

[22] Matsushita K, van der Velde M, Astor BC, Woodward M, Levey AS, de Jong PE, Coresh J, Gansevoort RT (2010). Association of estimated glomerular filtration rate and albuminuria with all-cause and cardiovascular mortality in general population cohorts: a collaborative meta-analysis. Lancet.

[23] van der Velde M, Matsushita K, Coresh J, Astor BC, Woodward M, Levey A, de Jong P, Gansevoort RT, van der Velde M, Matsushita K, Coresh J, Astor BC, Woodward M, Levey AS, de Jong PE, Gansevoort RT, Levey A, El-Nahas M, Eckardt KU, Kasiske BL, Ninomiya T, Chalmers J, Macmahon S, Tonelli M, Hemmelgarn B, Sacks F, Curhan G, Collins AJ, Li S, Chen SC, Hawaii Cohort KP, Lee BJ, Ishani A, Neaton J, Svendsen K, Mann JF, Yusuf S, Teo KK, Gao P, Nelson RG, Knowler WC, Bilo HJ, Joosten H, Kleefstra N, Groenier KH, Auguste P, Veldhuis K, Wang Y, Camarata L, Thomas B, Manley T (2011). Lower estimated glomerular filtration rate and higher albuminuria are associated with all-cause and cardiovascular mortality. A collaborative meta-analysis of high-risk population cohorts. Kidney Int.

[24] Kottgen A, Russell SD, Loehr LR, Crainiceanu CM, Rosamond WD, Chang PP, Chambless LE, Coresh J (2007). Reduced kidney function as a risk factor for incident heart failure: the atherosclerosis risk in communities (ARIC) study. J Am Soc Nephrol.

[25] McGonigle RJS, Fowler MB, Timmis AB (1984). Uremic cardiomyopath: potential role of vitamin D and parathyroid hormone. Nephron.

[26] Hawley CM, Holt SG (2017). Parathyroid hormone targets in chronic kidney disease and managing severe hyperparathyroidism. Nephrology.

[27] van Ballegooijen AJ, Reinders I, Visser M, Brouwer IA (2013). Parathyroid hormone and cardiovascular disease events: a systematic review and meta-analysis of prospective studies. Am Heart J.

[28] Aggarwal H, AlJaroudi WA, Mehta S, Mannon R, Heo J, Iskandrian AE, Hage FG (2014). The prognostic value of left ventricular mechanical dyssynchrony using gated myocardial perfusion imaging in patients with end-stage renal disease. J Nucl Cardiol.

[29] Malik D, Mittal BR, Sood A, Sharma A, Parmar M, Kaur K, Bahl A (2019). Evaluation of left ventricular mechanical dyssynchrony with phase analysis in end-stage renal disease patients with normal gated SPECT-MPI. World J Nucl Med.

[30] Rami D, Ibtihaj F, Tania C (2017). The prognostic value of regadenoson SPECT myocardial perfusion imaging in patients with end-stage renal disease. J Nucl Cardiol.

[31] Miller Erica O, Schwartz Ronald G (2017). Cardiovascular risk assessment with regadenoson SPECT MPI in patients with end-stage renal disease is safe, effective, and well tolerated: does it matter?. J Nucl Cardiol.

[32] Golzar Y, Doukky R (2017). Stress SPECT Myocardial perfusion imaging in end-stage renal disease. Curr Cardiovasc Imaging Rep.

[33] Burt JR, Zimmerman SL, Kamel IR, Halushka M, Bluemke DA (2014). Myocardial T1 mapping: techniques and potential applications. Radiographics.

[34] Taylor AJ, Salerno M, Dharmakumar R, Jerosch-Herold M (2016). T1 mapping: basic techniques and clinical applications. JACC Cardiovasc Imaging.

[35] Kellman P, Hansen MS (2014). T1-mapping in the heart: accuracy and precision. J Cardiovasc Magn Reson.

[36] Moon JC, Treibel TA, Schelbert EB (2013). T1 Mapping for diffuse myocardial fibrosis. J Am Coll Cardiol.

[37] Hayer MK, Radhakrishnan A, Price AM, Baig S, Liu B, Ferro CJ, Captur G, Townend JN, Moon JC, Edwards NC, Steeds RP (2019). Early effects of kidney transplantation on the heart. A cardiac magnetic resonance multi-parametric study. Int J Cardiol.

[38] Amann K, Ritz E, Wiest G, Klaus G, Mall G (1994). A role of parathyroid hormone for the activation of cardiac fibroblasts in uremia. J Am Soc Nephrol.

[39] Lekawanvijit S (2018). Cardiotoxicity of uremic toxins: a driver of cardiorenal syndrome. Toxins (Basel).

[40] Asche CV, Marx SE, Kim J (2012). Impact of elevated intact parathyroid hormone on mortality and renal disease progression in patients with chronic kidney disease stages 3 and 4. Curr Med Res Opin.

[41] Liu YW, Su CT, Huang YY (2011). Left ventricular systolic strain in chronic kidney disease and hemodialysis patients. Am J Nephrol.

